# The United States dermatology inpatient workforce between 2013 and 2019: a Medicare analysis reveals contraction of the workforce and vast access deserts—a cross-sectional analysis

**DOI:** 10.1007/s00403-024-02845-0

**Published:** 2024-03-14

**Authors:** Jourdan A. Hydol-Smith, Matthew A. Gallardo, Abraham Korman, Lauren Madigan, Sabrina Shearer, Caroline Nelson, Kristopher Fisher, Kalyn Hoffman, Arturo Dominguez, Benjamin H. Kaffenberger

**Affiliations:** 1 Texas A&M School of Medicine, Bryan, TX USA; 2 The Ohio State University College of Medicine, Columbus, OH USA; 3 Department of Dermatology, The Ohio State University Wexner Medical Center College of Medicine, 1328 Dublin Rd. Suite #100, Columbus, OH 43201 USA; 4 Department of Dermatology, University of Utah, Salt Lake City, UT USA; 5 Department of Dermatology, Duke University School of Medicine, Durham, NC USA; 6 Department of Dermatology, Yale School of Medicine, New Haven, CT USA; 7 Department of Dermatology, University of Texas Southwestern Medical Center, Dallas, TX USA

**Keywords:** Inpatient dermatology, Medicare, Rural populations, Metropolitan populations, Medical dermatology, Geographic variation

## Abstract

While time spent practicing inpatient dermatology has decreased since the 1990s, less is known about the current state of inpatient dermatology. We describe the distribution and frequency of inpatient dermatology encounters servicing the United States Medicare population between 2013 and 2019. Cross-sectional analysis of publicly available inpatient Medicare Part B claims data from 2013 to 2019 was conducted. Main outcomes and measures were characteristics and trends of dermatologists performing inpatient encounters. Categorical variables were compared using *χ*^2^ analysis. Trends were analyzed for linearity using Pearson correlation coefficient. 782 physicians met inclusion criteria for inclusion. Dermatologists were more often male (56.5%), possessing allopathic Medical Doctorate (MD) (86.3%), and in metropolitan settings (98.2%). However, proportion of female inpatient dermatologists increased significantly (37.9% to 46.2%). Across rural and metropolitan practices, number of inpatient physicians (2013: 356; 2019: 281) and number of medical centers in which dermatology encounters occurred (2013: 239; 2019: 157) decreased, more significantly in non-residency-associated institutions. Spatial analysis revealed wide regions lacking dermatologists meeting defined criteria. Limitations included the need for ten Medicare inpatient encounters for inclusion, counties without reported data. In conclusion, the number of dermatologists performing > 10 inpatient encounters per year is decreasing, and large variations exist in the number of U.S. inpatient dermatology visits.

## Introduction

Between 2010 and 2014, over 3.5 million hospitalizations occurred for all dermatologic diagnoses based on the nationwide readmissions database, with $508 million per year spent on same-cause readmission [1]. Numerous studies—particularly with regard to the diagnosis of cellulitis—have demonstrated a lack of consensus between primary admitting teams and dermatology consultants leading to significant differences in treatment and outcomes [2–4]. Proficient treatment of these patients is best done by dermatologists who have specific expertise in the diagnosis and management of patients with complex medical and socioeconomic comorbidities [2, 5].

Studies have demonstrated improved outcomes in hospitalized patients receiving dermatologic care, and in response, the Society of Dermatology Hospitalists (SDH) was formed [3, 6]. Despite improvement in outcomes, however, dedication to the practice of hospital dermatology remains rare among graduating dermatology residents [7, 8]. Although multiple studies have characterized contemporary practice patterns and landscape of outpatient dermatology, data are needed in the field of hospital dermatology [7–9]. This cross-sectional study was intended to directly characterize the distribution and characteristics of inpatient dermatologists in the United States.

## Materials and methods

We conducted a cross-sectional analysis of physicians who billed Medicare for inpatient dermatology encounters in the United States between January 2013 and December 2019. The analysis was done using published payment data from the Medicare Provider Utilization and Payment Data: Physician and Other Practitioners dataset, provided by the Centers for Medicare and Medicaid Services (CMS) (URL: https://data.cms.gov/provider-summary-by-type-of-service/medicare-physician-other-practitioners/medicare-physician-other-practitioners-by-provider-and-service/data/2013) [10]. This is a publicly available data set inclusive of physicians who have billed Medicare for 11 or more services in a calendar-year; those billing 10 or less are excluded to prevent patient identification. We first identified all dermatologists in the dataset based on the variable “Provider Type of the Provider.” We further stratified the dataset by dermatologists performing inpatient encounters using Current Procedural Terminology [CPT] codes. Codes 99221–99223 were used for initial inpatient encounters, and codes 99231–99233 were used for subsequent inpatient encounters.

Physician characteristics included gender, credentials, location of practice, and practice (urban or rural) setting. Physician credentials were stratified into Doctor of Medicine [MD], Doctor of Osteopathy [DO], and MD with an advanced degree. In our cohort, there were no DO physicians with an additional advanced degree performing inpatient dermatology encounters. Location of practice was stratified by census regions and divisions of the United States. Lastly, urban or rural practice setting was identified by first matching the physician’s practice ZIP code to state and county FIPS codes, using data from the SASHELP.ZIPCODE dataset [11]. Counties were then categorized as rural based on FIPS codes from the Consumer Financial Protection Bureau Rural Counties dataset [12].

Statistical analyses were performed in SAS version 9.4 software (SAS Institute, Cary, NC). The frequency and proportion of physician characteristics were tabulated annually. Categorical variables were compared using *χ*^2^ analysis. Unique institutions were identified by street address. Lastly, a map of the United States was constructed based on the mean number of inpatient encounters in each county over the 7-year period.

## Results

We identified 782 unique physicians who met the inclusion criteria (Table 1). This included 356 physicians in 2013 and decreased steadily to 281 physicians by 2019. Dermatologists who performed inpatient encounters over the 7-year period were more often male (56.5%), and more than 80 percent possessed only an MD degree (86.3%). Most dermatologists performing inpatient encounters were located in the Northeast (32.9%), South (31.2%), and Midwest (25.7%). Lastly, most inpatient dermatology encounters took place in a metropolitan practice setting (98.2%). Based on *χ*^2^ analysis, physician credentials, location of practice, and county classification showed no significant change between 2013–2015 and 2017–2019. However, the proportion of female dermatologists caring for hospitalized patients increased significantly from 37.9% between 2013 and 2015 to 46.2% between 2017 and 2019 (*p* = 0.008).

**Table 1:** Physician demographics

	2013–2015	2017–2019	2013–2019	*p* value*
*N* dermatologists conducting inpatient consults	531	450	782	
Gender				0.008
Male	330 (62.1%)	242 (53.8%)	442 (56.5%)	
Female	201 (37.9%)	208 (46.2%)	340 (43.5%)	
Physician credentials				0.811
MD**	458 (86.3%)	388 (86.2%)	675 (86.3%)	
DO***	25 (4.7%)	18 (4.0%)	33 (4.2%)	
MD+ advanced degree	48 (9.0%)	44 (9.8%)	74 (9.5%)	
Location of practice				0.242
West	52 (9.8%)	50 (11.1%)	80 (10.2%)	
Midwest	133 (25.1%)	125 (27.8%)	201 (25.7%)	
South	178 (33.5%)	124 (27.6%)	244 (31.2%)	
Northeast	168 (31.6%)	151 (33.5%)	257 (32.9%)	
County classification				0.738
Metropolitan	523 (98.5%)	442 (98.2%)	768 (98.2%)	
Rural	8 (1.5%)	8 (1.8%)	14 (1.8%)	

Categorical variables as number (percentage)

^*^p values are comparing change from 2013–2015 to 2017–2019

^**^Doctor of Allopathic Medicine

^***^Doctor of Osteopathic Medicine

**Fig. 1:**
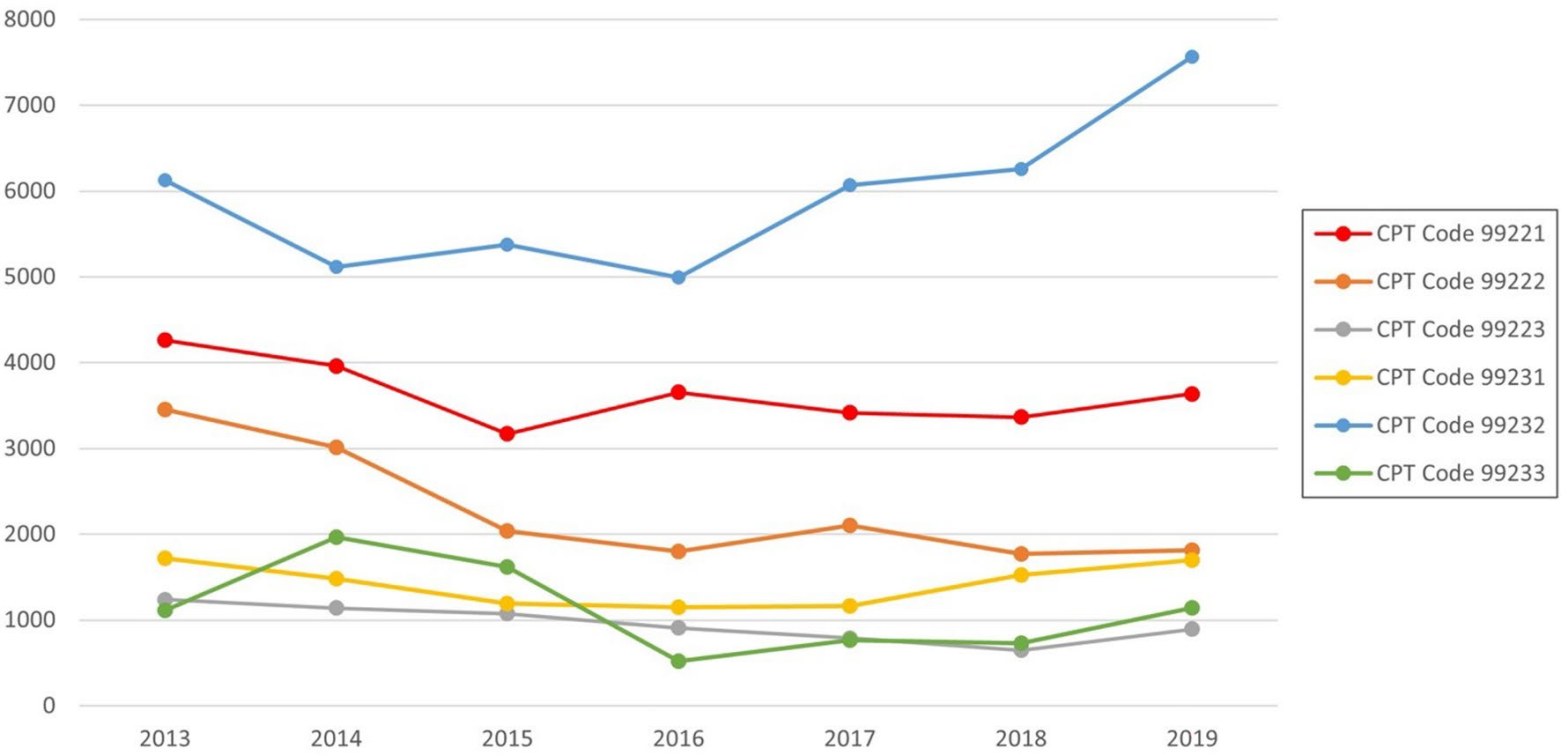
Total number of inpatient dermatology encounters from 2013 to 2019 with dermatology encounter CPT codes

**Fig. 2:**
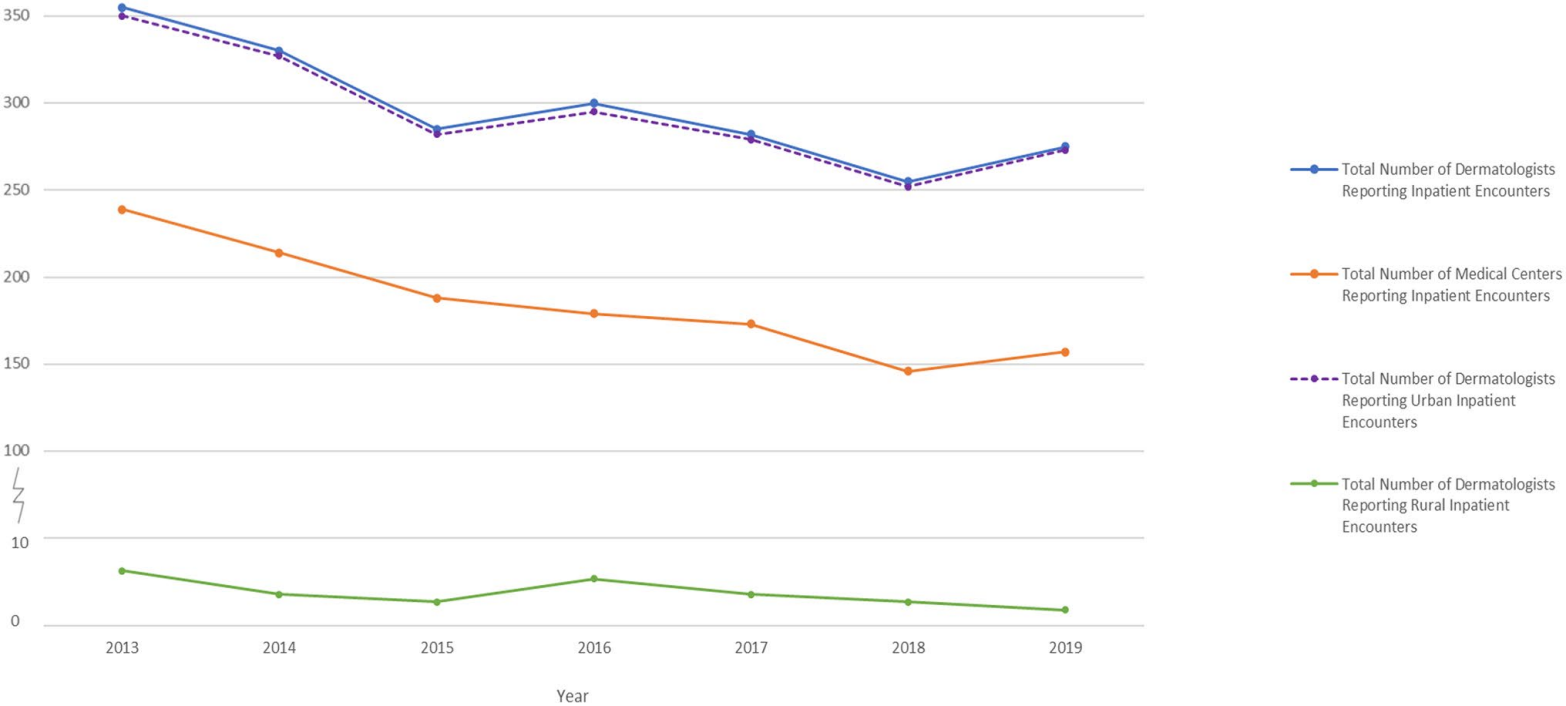
Characterization of volume and location of inpatient dermatology encounters in the United States Medicare Population between 2013 and 2019

The total number of inpatient dermatology encounters (Fig. 1) decreased from 2013–2019 (*r* = − 0.368, *p* value = 0.417), however, at a much lower rate compared to the number of inpatient dermatology encounter-providing dermatologists, and the number of medical centers providing dermatology encounters. Inpatient dermatology encounter-providing dermatologists (Fig. 2) decreased from 2013 to 2019 (*r* = − 0.836, *p* value = 0.019), in both rural (*r* = − 0.696, *p* value = 0.082) and metropolitan practices (*r* = − 0.831, *p* value = 0.020). Similarly, the number of medical centers providing dermatology encounters (Fig. 2) decreased from 2013 to 2019 (*r* = − 0.948, *p* value = 0.001).

**Fig. 3:**
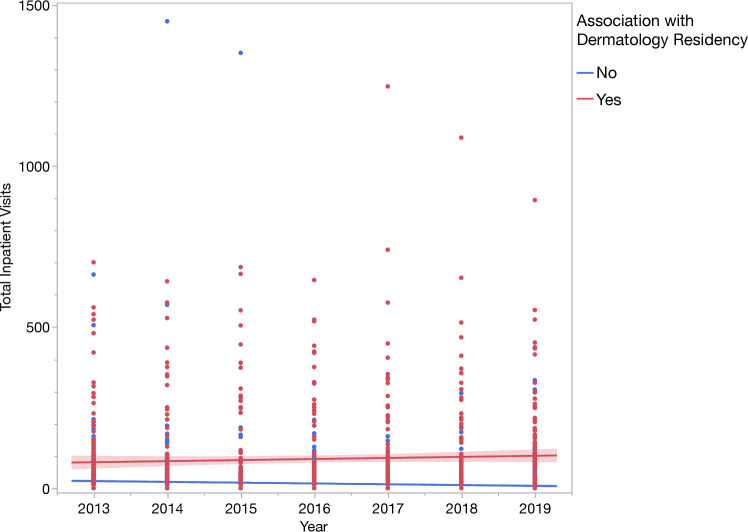
Line of best fit for total inpatient visits from 2013 to 2019, stratified by hospitals with and without an Associated Dermatology Residency Program

Combination of associated institution addresses identified 408 unique institutions performing inpatient dermatology consults from 2013 to 2019, of which 100 (24.5%) were affiliated with a dermatology residency program. One non-residency-associated institution was a clear statistical outlier and another address could not be associated with an institution, so these were excluded from further analysis. Regression modeling revealed that in 2013, the average predicted residency-associated program was conducting 26 more inpatient consults than the average non-residency-associated institution. This difference in predicted inpatient consults between residency-associated programs and non-residency-associated programs grew by 5.8 consults for each year of the model through 2019. This initial difference in consult number coupled with an increased rate of growth every year is clearly visualized (Fig. 3).

**Fig. 4:**
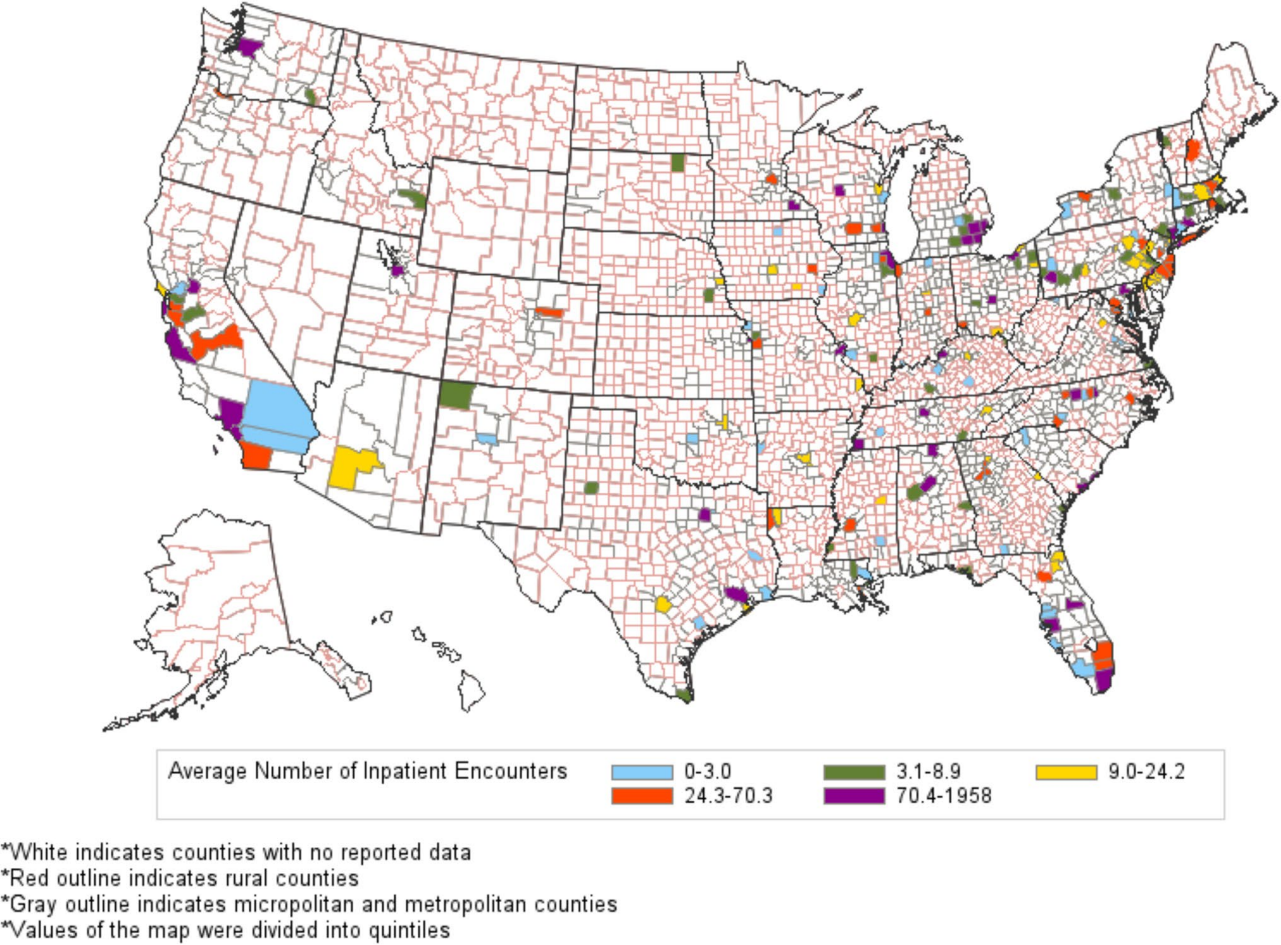
County-level map of the average number of yearly dermatology inpatient encounters in the United States Medicare Population from 2013 to 2019

Significant geographic variation was also noted when mapping the data onto a county-level map of the United States, confirming that inpatient dermatology access exists primarily within metropolitan regions with widespread areas lacking access. States completely lacking physicians meeting the inclusion criteria over the 7-year period include Alaska, Arkansas, Hawaii, Maine, Montana, Nevada, North Dakota, and Wyoming (Fig. 4).

## Discussion

Misdiagnoses of dermatologic conditions in medically complex patients may lead to longer admissions, potentially introducing harm [13]. To limit this occurrence, inpatient dermatologists with specific expertise in the investigation and management of skin disease in hospitalized patients are needed [3]. Our analysis demonstrates a linear decrease in the number of dermatologists performing inpatient encounters throughout the United States. Some of this change may be attributable to greater specialization, such as the emergence of dermatology hospitalists; however, the number of medical centers providing these inpatient encounters also decreased linearly over the same time-period. This finding is consistent with historical trends, including a prior survey of academic programs demonstrating a steady decrease in the number of inpatient dermatology encounters within academic medical centers and the nation as a whole [14, 15]. Based on these trends, the number of inpatient dermatologists is expected to further decrease over time despite the great need that exists within healthcare systems.

Dermatologists performing inpatient services are unevenly distributed across the country. While this finding is consistent with prior research, our study further demonstrates these geographic disparities at a more granular level, highlighting geographic care islands within each state [15]. Our analysis demonstrates that most dermatologists performing inpatient services are concentrated in large urban areas which likely relates to higher population densities and access to regional and referral centers. Urban inpatient dermatologists may also benefit from improved incentives and support from high-volume healthcare systems. The result is a significant gap in inpatient dermatologic care for rural populations, forcing complex dermatologic patients to travel to seek healthcare.

Our analysis further demonstrates a unique observation of the demographic data of dermatologists performing greater than ten inpatient encounters per year. Throughout the 7-year period, there was an overall increase in the percentage of female dermatologists performing inpatient encounters. This is likely related to the significant positive impact of mentorship among women pursuing dermatology, especially from dermatologists within the SDH who may inspire dermatology residents to pursue complex medical dermatology [16].

Notably, the small number of hospitals shown to conduct dermatologic consults appear to be seeing a greater absolute number of patients even with fewer dermatologists ultimately seeing Medicare patients in the hospital setting. Although it is possible this is due to fewer hospitalizations for skin disease, this likely supports greater specialization in the field and a greater propensity to transfer patients to regional referral centers to dermatology residency-associated programs, demonstrated by the much higher number and rate of growth of consults for dermatology residency-associated institutions compared to hospitals without a dermatology residency. Even among the non-residency affiliated programs, many are located in urban centers, further suggesting vast regions of the United States have inconsistent access to dermatology.

This study’s primary limitations include a reliance on Medicare billing data, restriction to physicians participating in Medicare, low number of rural dermatologists, and a lack of representation of low-volume consulting physicians (total services ≤ 10). Dermatologists performing less than ten inpatient encounters per year were not included in the Medicare Payment Data by Medicare for privacy concerns. Our analysis also only included Medicare data and does not include privately insured or uninsured patients. Additionally, our analysis likely does not include any pediatric patients given it is limit to the Medicare patient population only. Lastly, practice patterns may have changed within the last 3 years, especially during the COVID-19 pandemic.

## Conclusions

There are large regional variations in the number of inpatient dermatology encounters occurring across the United States, and an observed decrease in the number of physicians and medical centers performing inpatient dermatology encounters. [14, 17] Increased support for both new and existing hospital dermatologists and strategies to improve access to inpatient dermatologic care are necessary to minimize wide and worsening access disparities.

## Abbreviations

SDH: Society of Dermatology Hospitalists
CMS: Centers for Medicare and Medicaid Services
CPT: Current Procedural Terminology
MD: Doctor of Medicine
DO: Doctor of Osteopathy
